# A novel decomposer-exploiter interaction framework of plant residue microbial decomposition

**DOI:** 10.1186/s13059-025-03486-w

**Published:** 2025-02-03

**Authors:** Youzhi Miao, Wei Wang, Huanhuan Xu, Yanwei Xia, Qingxin Gong, Zhihui Xu, Nan Zhang, Weibing Xun, Qirong Shen, Ruifu Zhang

**Affiliations:** Jiangsu Provincial Key Lab of Solid Organic Waste Utilization, Jiangsu Collaborative Innovation Center of Solid Organic Wastes, Key Lab of Organic-Based Fertilizers of China, Educational Ministry Engineering Center of Resource-Saving Fertilizers, Nanjing Agricultural University, Nanjing, China

**Keywords:** Decomposer-exploiter, Fungi and bacteria, Plant residue decomposition, Soil organic carbon

## Abstract

**Background:**

Plant residue microbial decomposition, subject to significant environmental regulation, represents a crucial ecological process shaping and cycling the largest terrestrial soil organic carbon pool. However, the fundamental understanding of the functional dynamics and interactions between the principal participants, fungi and bacteria, in natural habitats remains limited.

**Results:**

In this study, the evolution of fungal and bacterial communities and their functional interactions were elucidated during the degradation of complexity-gradient plant residues. The results reveal that with increasing residue complexity, fungi exhibit heightened adaptability, while bacterial richness declines sharply. The differential functional evolution of fungi and bacteria is driven by residue complexity but follows distinct trajectories. Fundamentally, fungi evolve towards promoting plant residue degradation and so consistently act as the dominant decomposers. Conversely, bacteria predominantly increase expression of genes of glycosidases to exploit fungal degradation products, thereby consistently acting as exploiters. The presence of fungi enables and endures bacterial exploitation.

**Conclusions:**

This study introduces a novel framework of fungal decomposers and bacterial exploiters during plant residue microbial decomposition, advancing our comprehensive understanding of microbial processes governing the organic carbon cycling.

**Supplementary Information:**

The online version contains supplementary material available at 10.1186/s13059-025-03486-w.

## Background

Plant residue microbial decomposition (PRMD) is the pivotal driving force of the largest terrestrial soil organic carbon pool [1, 2]. Fungi and bacteria are the major participants in this decomposition process [3]. Therefore, understanding their roles and interactions in PRMD under natural conditions is critical for understanding the ecological framework of organic carbon cycling. In forest ecosystems, fungi predominantly govern the decomposition of plant residues such as litter and wood, as evidenced by their disproportionately high levels of fungal biomass accumulation, residue-degrading gene transcription, and related enzyme secretion [4–8]. However, recent studies have escalated attention towards bacteria, highlighting their underestimated yet vital role in the PRMD of forest ecosystems [9–11]. In the forest topsoil of the organic horizon, bacteria and fungi displayed comparable contributions to the transcription of plant residue-degrading genes [7]. Specifically, bacteria could substantially sequestering cellulosic and lignin carbon within their biomass across the litter and organic horizons of forest soil [12, 13]. In contrast to forest ecosystems, in grasslands and agroecosystems, which store over 40% of terrestrial carbon [14] and are highly sensitive to environmental perturbations, research on PRMD has mainly focused on bacterial processes. For example, bacterial keystone taxa have been shown to drive straw decomposition [15], with *Acidobacteria*, *Actinobacteria*, and *Proteobacteria* contributing critically to the transcription of plant residue-degrading genes [16, 17].

Fungi and bacteria both play crucial roles in PRMD, however, their functions vary markedly, and a comprehensive understanding of their unique contributions and interactions is lacking. Plant residue with high complexity, particularly lignin and crystalline cellulose, is a substantial barrier to microbial decomposition [18]. In forest ecosystems, high levels of these recalcitrant compounds prevent the breakdown of litter and wood, while in grasslands and agroecosystems, hemicellulose-rich annuals decompose more readily [19]. This distinction couples with fungal dominance in forest ecosystems and bacterial prominence in grasslands and agroecosystems, suggesting a dynamic contribution spectrum of fungi and bacteria in PRMD associated with the residue complexity. A rare aquatic residue decomposition simulation supports this model, where bacteria and fungi specialize in decomposing low (algae) and high-complexity (beech litter) residues, respectively [20]. Notably, the high C/N ratio in topsoil, usually due to plant residues with high complexity [21], increases the fungi-to-bacteria (F/B) ratio globally [22]. Moreover, high plant diversity not only elevates the F/B ratio [23] but also enhances the residue decomposition rate [24]. Clearly, plant diversity involves complexity-related structural variations in plant residues [25]. Therefore, plant residue complexity may drive the functional trade-off between fungi and bacteria across different habitats. Overall, shifts in global F/B ratios imply cross-kingdom interactions [26], and the intricate decomposition roles likely result from complex fungi-bacteria interplay modulated by residue complexity.

The understanding of multispecies interactions in complex environments is limited, although the “exploiter-cooperator” framework provides primary insights within bacterial groups. In this framework, cooperators produce extracellular public goods to capture scarce resources, whereas exploiters pilfer these public goods, and deceitfully rely on cooperators for survival [27]. System balance largely depends on the effectiveness and severity of the punishment deployed by cooperators against exploiters [28, 29]. Lignocellulosic enzymes involved in plant residue decomposition exhibit strong public goods characteristics. Consequently, the following questions arise: In PRMD, how do these microbes modulate their enzyme-mediated decomposition roles? Do they cooperate, compete, or engage in complex, higher-order interactions? A comprehensive understanding of these questions is essential.

In this study, the microbial evolutionary processes in plant residue decomposition were elucidated, and the results revealed that residue complexity drives the trade-off of fungal and bacterial interactions. Bacteria consistently evolve as exploiters, while fungi serve as decomposers for bacterial exploitation. Our findings provide novel insights into the interactions and roles of fungi and bacteria, contributing to the comprehensive understanding of the core ecological process of PRMD.

## Results

### Plant residue complexity drives the differentiation of lignocellulose-decomposing microbial communities

**Fig. 1:**
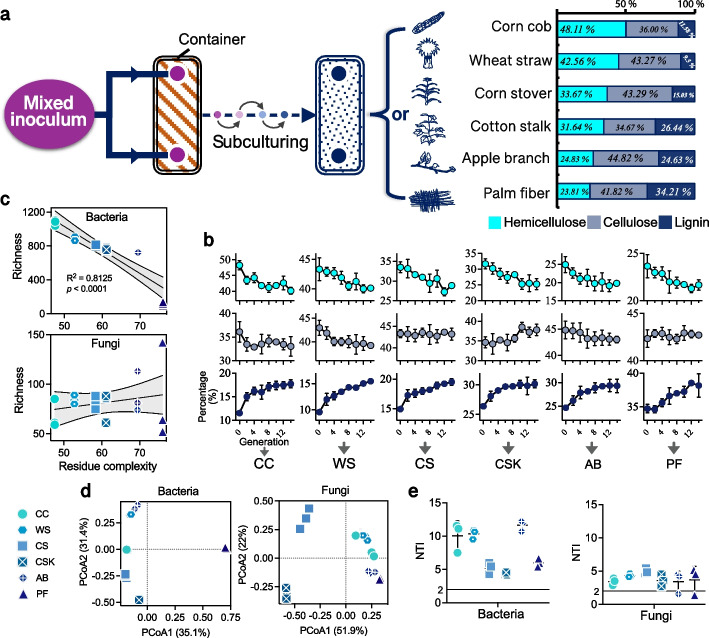
Microbial evolutionary experiment across complexity-gradient plant residues. A mixed microbial inoculum was subjected to successive solid-phase subculturing (total 14 generations, 15 days each) using the substrate of each plant residue—corn cob (CC), wheat straw (WS), corn stover (CS), cotton stalk (CSK), apple branch (AB), and palm fiber (PF)—possessing a gradient change in cellulose, hemicellulose, and lignin contents (**a**). The dynamic changes of these polymers, in response to the microbial degradation after each generation of incubation, were determined (**b**). The 16S rDNA and ITS samples at the 14th generation were sequenced for bacterial and fungal communities, respectively. Thus, the richness index (**c**), Bray–Curtis-based principal coordinate analysis (PCoA) (**d**), and the nearest taxon index (NTI) analysis (**e**) were performed. Each sample included three biological replicates

To investigate the evolution of the decomposing microbial community in different plant residues, six types of plant residues were inoculated with the same microbial consortia and subjected to fourteen successive subcultures of solid fermentation (Fig. 1a). The initial consortia, homogenized from 127 decomposition-associated environmental samples (see the “Methods” section), were prepared to capture diverse potential decomposing microorganisms from natural habitats. The plant residues include corn cob (CC), wheat straw (WS), corn stover (CS), cotton stalk (CSK), apple branch (AB), and palm fiber (PF) with gradient-varying cellulose, hemicellulose, and lignin contents (Fig. 1a), representing different levels of complexity. During this process, the degradation capacity gradually increased until reaching a stable level in approximately the eighth subculturing generation (Fig. 1b). Interestingly, in the evolved decomposing microbial communities, the bacterial richness was negatively correlated with the plant residue complexity (Fig. 1c, *R*^2^ = 0.81, *p* < 0.0001), while the fungal richness was not significantly affected and even slightly increased with the increasing complexity of plant residues. The dissimilarity within the bacterial and fungal communities suggests that they evolved in distinct manners (Fig. 1d). The nearest taxon indices of the communities (NTI > 2) demonstrated highly variable selection processes (Fig. 1e), indicating that plant residue complexity dominated the assembly of microbial communities. These results suggest that fungi, with their outstanding and widespread decomposition abilities, may serve as potential decomposers in plant residue decomposition. In contrast, the scarcity of such abilities within bacterial communities may induce a dramatic species elimination with increasing plant residue complexity.

### Fungi dominate CXL genes, while bacteria enrich OH and PBH genes in the decomposing microbial communities

**Fig. 2:**
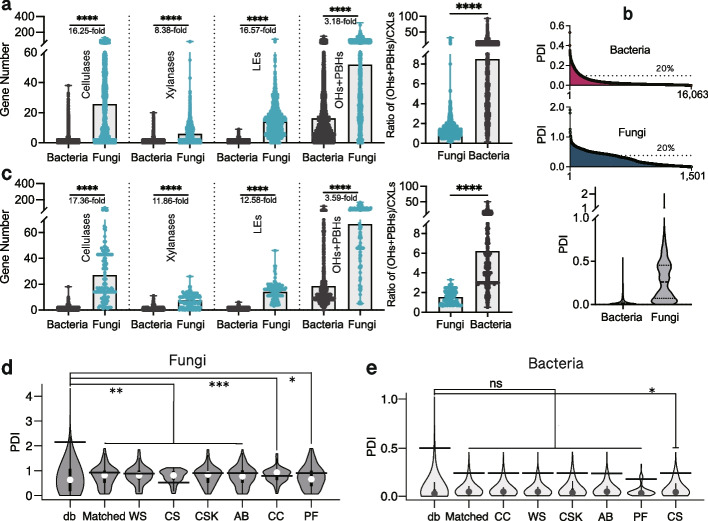
Genomic analysis of bacterial and fungal CAZymes involved in plant residue decomposition. **a** The gene copy number of cellulase, xylanase, ligninolytic enzyme (LE), OH and PBH, and the ratio of OHs and PBHs to CXLs (cellulases, xylanases, and ligninolytic enzymes) in each genome from the 16,063 bacterial and 1501 fungal non-redundant genomes. Bar graphs represent gene averages per genome and points represent individual genomes. **b** The plant residue decomposition index (PDI, see the “Methods” section) distribution of the non-redundant genomes (20% represents the location of the 20% of the highest PDI value). **c** The gene copy number of cellulase, xylanase, ligninolytic enzyme (LE), OH and PBH, and the ratio of OHs and PBHs to CXLs (cellulases, xylanases, and ligninolytic enzymes) in each genome from the matched 1538 bacterial and 126 fungal genomes. The PDI distribution in each evolved fungal (**d**) and bacterial (**e**) community is shown. Semi-range values are indicated by horizontal lines. “db” represents the non-redundant fungal/bacterial genomes; “Matched” represents the OTU-matched fungal/bacterial genomes. Asterisks denote statistical significance: *, *p* < 0.05; **, *p* < 0.01; ***, *p* < 0.001; ****, *p* < 0.0001. ns, not significant

To reveal the driving factor by which the highly complex plant residues restrict the bacterial richness rather than the fungal richness, we first compared their potential from a genomic perspective. A total of 16,063 bacterial and 1501 fungal non-redundant genomes were collected from both the NCBI and JGI databases, and a comprehensive annotation and analysis of carbohydrate-active enzymes (CAZymes) was performed (Additional file 1). Microbial cellulases and xylanases hydrolyze the predominant cellulose and xylan polysaccharides in plant residues. Genome-wide statistical analyses revealed that the bacterial genomes contain 1.59, 0.72, and 0.86 genes on average that encoded for cellulases, xylanases, and ligninolytic enzymes (collectively referred to as CXLs), respectively. Unsurprisingly, the fungal genomes contain 16.5- (25.84), 8.38- (6.04), and 16.57-fold (14.25) more encoding genes, respectively, than the bacterial genomes (Fig. 2a and Additional file 1). Unlike the CXL genes, bacterial genomes contain more genes (16.36 per genome) encoding oligosaccharide hydrolases (OHs) and polysaccharide-branch hydrolases (PBHs) (Fig. 2a and Additional file 1). Moreover, bacterial genomes enriched more OH and PBH genes than fungi, as evidenced by the significantly higher (OHs + PBHs)/CXLs ratio (Fig. 2a).

To estimate the plant residue-degrading potentials of each bacterium and fungus, we developed a weighted ranking value of the plant residue decomposition index (PDI) based on the quantity and diversity of the CAZymes in each genome (see the “Methods” section). The average PDI of fungi was 7.96 times higher than that of bacteria, and fungal PDI distribution exhibited a higher uniformity, with fungi (36.1%) being more prevalent in high PDI values (≥ 20% PDI_max_) compared to bacteria (8.5%) (Fig. 2b and Additional file 1). The above results suggest the great and universal plant residue-degrading potential of fungi. Then, to evaluate the plant residue-degrading potential of the evolved decomposing microbial communities after solid fermentation subculturing, the detected bacterial and fungal operational taxonomic units (OTUs) were matched with the non-redundant genomes using a stringent sequence similarity criterion (see the “Methods” section). These matched OTUs represent 70.2% (1538/2190) and 40.8% (126/309) of the detected bacteria and fungi, accounting for 92.8% and 93.5% of the sequences, respectively (Additional file 2). The matched fungal genomes exhibit 17.36-, 11.86-, and 12.58-fold higher levels of cellulases-, xylanases-, and ligninolytic enzyme-encoding genes per genome, respectively, than the matched bacterial genomes (Fig. 2c). Moreover, the matched bacterial genomes show a higher proportion of OHs and PBHs (18.59 genes per genome) than the CXLs (1.11 genes per genome) (Fig. 2c). Significant enrichment of genomes possessing high PDI values was observed in the evolved fungal communities, with an average of 50.78% PDI values exceeding semi-range (half of the max–min range) while only 0.53% in the 1501 fungal genomic pool (Fig. 2d), revealing that fungal communities have evolved to promote plant residue degradation. However, similar enrichment did not occur in those of bacterial communities, showing no divergence in PDI distribution from the 16,063 bacterial genomes pool (Fig. 2e). Therefore, fungi have great plant residue decomposition potential with a high abundance of CXL genes, whereas bacteria are most likely to engage in opportunistic competition for the products released from plant residue decomposition when harboring many OH- and PBH-encoding genes but few CXL genes. Specifically, over 76% of the bacteria (relative abundance) contain fewer than two cellulase- and/or xylanase-encoding genes, whereas only 7.1% of the fungi contain fewer than eight cellulase- and/or xylanase-encoding genes (Additional file 2). These results indicate that the fungal communities with high plant residue-degrading capability were enriched in different plant residues rather than the bacterial communities. Surprisingly, although the bacterial richness was significantly decreased, the bacterial communities were always dominated by exploiters (with negligible or minimal plant residue decomposition capability but strong product competitiveness) than the expected decomposers (relatively high plant residue decomposition capability) with increasing plant residue complexity.

### Fungi are primary decomposers, while bacteria are exploiters in plant residue decomposition

**Fig. 3:**
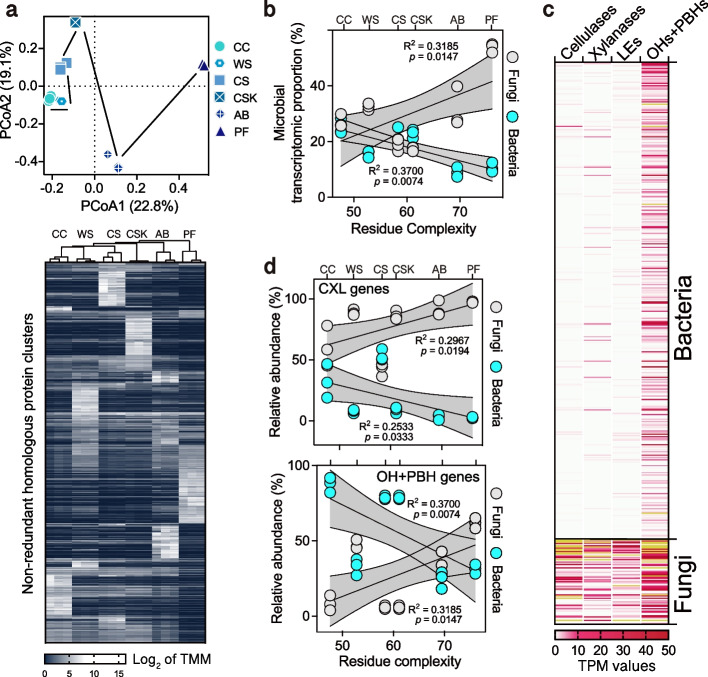
Metatranscriptomic analysis of bacteria and fungi in plant residue decomposition. **a** PCoA analysis and heatmap for expression changes of the top 9500 non-redundant protein clusters (see the “Methods” section) in the metatranscriptomic samples after 14 generations of incubation. CC, WS, CS, CSK, AB, and PF represent each plant residue-incubated metatranscriptomic sample, respectively, with three biological replicates. The color key represents the range from the lowest (navy blue) to the highest (white) log_2_-transformed TMM values. **b** The relationship between residue complexity and the transcriptomic proportion of bacteria and fungi in the metatranscriptomic samples. Approximately 60.1% of non-GT family CAZymes identified in the metatranscriptomic samples were matched to 485 non-redundant OUT-matching bacterial and fungal genomes (bacteria: 411, fungi: 74). **c** Total TPM values of the aligned cellulase, xylanase, ligninolytic enzyme, and OH + PBH genes across all metatranscriptomic samples for each genome. The color key represents the range from the lowest (white) to the highest (burgundy) TPM values, with any values exceeding 50 indicated in yellow. **d** The relationship between residue complexity and the expression of CXL (cellulases, xylanases, and ligninolytic enzymes) genes (top) and the expression of OH + PBH genes (bottom)

To further clarify the fungal and bacterial contributions to plant residue decomposition, the metatranscriptomes of the solid fermentation samples of the fourteenth subculturing were detected. A total of 3,910,074 nucleic acid sequences were assembled and 2,703,740 proteins (≥ 80 AA) were annotated, of which 969,336 (35.85%) and 490,173 proteins (18.13%) were derived from bacteria and fungi, respectively (Additional file 3: Fig. S1). Using CD-HIT (≥ 40% sequence identity, ≥ 80% sequence coverage), all annotated proteins were clustered into 862,953 non-redundant homologous protein clusters (Additional file 3: Fig. S1), representing the overall biological functions in microbial communities. The functional dissimilarity increased with increasing residue complexity (Fig. 3a and Additional file 3: Fig. S2), confirming the crucial role of residue complexity in shaping the functional structure of microbial communities. Interestingly, the residue complexity and the proportion of bacterial transcriptional sequences were negatively correlated (Fig. 3b, *R*^2^ = 0.37, *p* < 0.01). In contrast, the proportion of fungal transcriptional sequences was positively correlated with the residue complexity (Fig. 3b, *R*^2^ = 0.32, *p* < 0.05). This suggests that increasing residue complexity resulted in higher survival pressure on bacteria, providing further evidence for the high adaptability of fungi to complex plant residues.

Further analysis identified a total of 12,813 non-glycosyl transferase (non-GT) family CAZymes from the metatranscriptomes, including 4654 fungal proteins and 5452 bacterial proteins (Additional file 3: Fig. S3 and Additional file 4). Approximately 60.1% (7702/12,813) of the proteins could be assigned to the represented genomes of bacterial and fungal OTUs (Additional file 2). From this matching, the total transcriptional abundances of cellulases, xylanases and ligninolytic enzymes were 48.5-, 13.5-, and 27.0-fold higher in fungi than in bacteria (Fig. 3c). However, the total transcriptional abundance of OHs and PBHs in bacteria was comparable to that in fungi and 31.4-fold higher than that of bacteria’s own CXLs (Fig. 3c).

Other organisms (including nematodes, protozoans, archaea, etc.), with transcriptional proportions varying between 45.7 and 63.5% (Fig. 3b), contributed only 5.7%, 4.3%, and 3.9% to the total transcriptional abundance of cellulases, xylanases, and ligninolytic enzymes, respectively (Additional file 4). This suggests that despite only 60.1% non-GT CAZymes match between metatranscriptomic and amplicon data, our exclusive focus on fungi and bacteria was scientifically justifiable in the context of plant residue decomposition. Consequently, analysis of all non-GT CAZymes data in metatranscriptomes revealed a significant positive correlation between residue complexity and the expression of fungal CXL genes (Fig. 3d, *R*^2^ = 0.30, *p* < 0.05; Additional file 3: Fig. S4), while a significant negative correlation with bacterial CXL genes (Fig. 3d, *R*^2^ = 0.25, *p* < 0.05; Additional file 3: Fig. S4). Importantly, in highly complex plant residues (WS, CSK, AB, and PF), fungi contributed to over 90% of CXL gene transcription (Fig. 3d). In contrast, bacteria exhibited a competitive advantage in the hydrolysis of oligosaccharides and polysaccharide-branches (Fig. 3d) despite their significant low genomic OH and PBH gene copies compared to fungi (Fig. 2a). Bacterial OHs and PBHs had 79–87% abundances in low-complexity plant residues (CC and CS), and still accounted for 25–31% abundances in high-complexity plant residues (AB and PF) (Fig. 3d) in stark contrast to only 2% abundance of their CXL genes.

Taken together, these results provide compelling evidence supporting that fungal communities dominated the plant residue decomposition. Meanwhile, increasing complexity of plant residues sharply decreased the decomposition capability of bacterial communities. However, bacterial exploiters, the free-rider consuming the decomposition products of fungi, always dominated the bacterial communities.

### Bacterial richness decline was associated with the available carbon types and concentrations

**Fig. 4:**
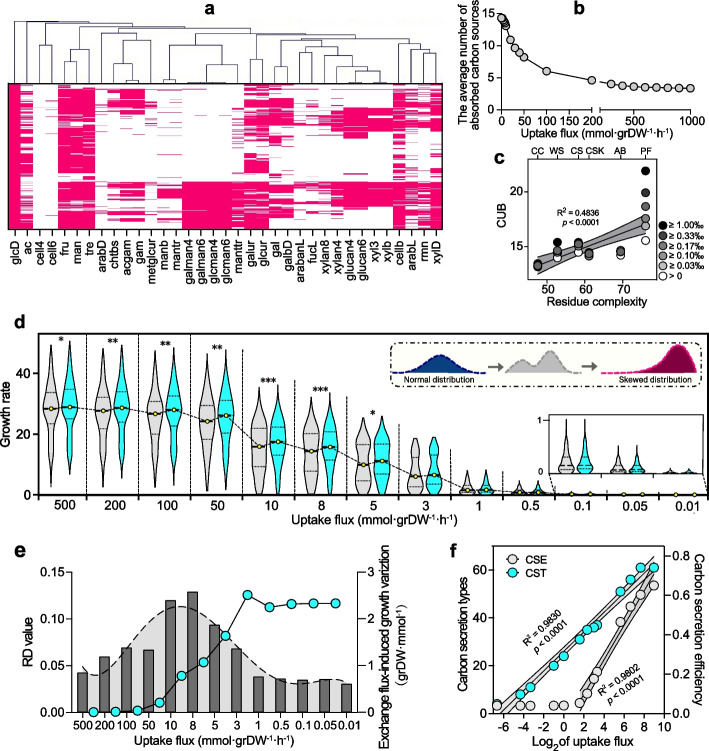
FBA analysis of the 421 bacterial GMMs. **a** The capability of the GMMs to utilize 35 plant residue-derived carbon sources (red represents available and white represents unavailable). The horizontal axis represents different carbon sources (Additional file 5), and the vertical axis represents different GMMs. **b** The variation in the average number of absorbed carbon sources per GMM across different uptake fluxes using a mixed medium with 35 carbon sources. **c** The relationship between residue complexity and the average carbon utilization breadth (CUB) value of each evolved bacterial community (CC, WS, CS, CSK, AB, and PF), calculated using the OTUs above different abundances from 0 to 1‰, respectively. **d** The growth rate dynamics of each bacterial GMM in the community across different uptake fluxes, without considering (gray violin plots) or considering (turquoise violin plots) cross-feeding interactions (see the “Methods” section). **e** The relative divergence (RD) of community growth rates induced by cross-feeding (dark gray histograms) and the variability of community growth rates (turquoise dot plots). **f** The relationship between the log_2_-transformed uptake flux and the carbon secretion type (CST) and efficiency (CSE)

To address what triggered the dramatic loss of bacterial richness with the increased plant residue complexity, we collected all 421 non-redundant bacterial genomes from the 1538 detected and genome-matched bacterial OTUs and then constructed all genome-scale metabolic models (GMMs) for these genomes. The simulation results indicate significant differences for each GMM to utilize the 35 carbon sources, which were the possible decomposition products of the plant residue components (Additional file 5). We observed that the carbon utilization breadth (CUB, the number of utilized carbon sources) of these bacteria ranged from 1 to 32 (Fig. 4a). Then, the flux balance analysis (FBA) was performed for all 421 GMMs in a medium containing all these 35 carbon sources with different concentrations. Under non-limiting conditions (uptake flux > 200 mmol·grDW^−1^·h^−1^ for each carbon source), these bacteria used an average of 3.5 carbon sources. However, the used carbon sources were increased to 14.3 when the uptake flux was declined to almost 0 mmol·grDW^−1^·h^−1^ (Fig. 4b), which highlighted the crucial role of CUB in bacterial survival under low-carbon conditions. Interestingly, the CUB values of the bacterial communities from different plant residues were positively correlated (Fig. 4c, R^2^ = 0.48, *p* < 0.0001) with residue complexity, and they were significantly higher than those of randomly chosen bacteria from the 16,063 genomes pool (Additional file 3: Fig. S5). Therefore, CUB was probably a key factor affecting bacterial adaptation in environments where high-complexity plant residues limited the carbon sources.

Under sufficient carbon sources (uptake flux > 10 mmol·grDW^−1^·h^−1^), bacterial growth rates follow a unimodal shape (normal distribution) (Fig. 4d, gray). However, their growth rates were shifted to a bimodal distribution and to a negative skewness distribution with the decreasing of carbon concentration (10 mmol·grDW^−1^·h^−1^ to 0.01 mmol·grDW^−1^·h^−1^) (Fig. 4d, gray), suggesting that only a few bacteria exhibited a relatively high growth rate, while most were inhibited. Notably, this distributional shift evidently impacted bacterial stability, thereby accounting for the decline in richness. However, the situation may have been more complex; in bacterial communities, cross-feeding mechanisms are prevalent, which sustain or even enhance their survival. To evaluate how cross-feeding affects the bacterial growth, we further simulated the 421 GMMs utilizing 35 plant residue-derived and up to 71 bacteria-secreted carbon sources (the secreta of 421 GMMs when simulated using 35 plant residue-derived carbon sources, Additional file 5). Considering the cross-feeding effects, the overall growth rates of the bacterial community were significantly increased when the uptake flux was above 5 mmol·grDW^−1^·h^−1^, which delayed the unimodal to bimodal distribution transition under 10 to 3 mmol·grDW^−1^·h^−1^ (Fig. 4d, turquoise). These results suggest that cross-feeding was beneficial for maintaining bacterial growth under carbon-restricted conditions. However, cross-feeding could not prevent the eventual decline in growth rates or the distribution transition (Fig. 4d, uptake flux < 10 mmol·grDW^−1^·h^−1^), although the cross-feeding efficiency was peaked during intervals of sharp declines in growth rates (Fig. 4e, 10 mmol·grDW^−1^·h^−1^ to 3 mmol·grDW^−1^·h^−1^). Indeed, both carbon secretion efficiency (CSE, *R*^2^ = 0.9830, *p* < 0.0001) and carbon secretion types (CST, *R*^2^ = 0.9802, *p* < 0.0001) were positively correlated with uptake flux (Fig. 4f). Particularly, when the uptake flux was lower than 3 mmol·grDW^−1^·h^−1^, the bacterial CSEs were declined to zero (Fig. 4f), indicating the near absence of secretion and cross-feeding. Therefore, the mitigative effect of cross-feeding on bacterial growth limitation was negligible under low carbon concentrations (< 3 mmol·grDW^−1^·h^−1^), although the cross-feeding efficiency was prominent. Collectively, under carbon-limited conditions with increasing plant residue complexity, the dramatic decline in bacterial growth rates and the negative skewness distribution without cross-feeding explained why there was a dramatic loss of bacterial richness.

### The presence of fungal decomposers induced the prevalence of bacterial exploiters

**Fig. 5:**
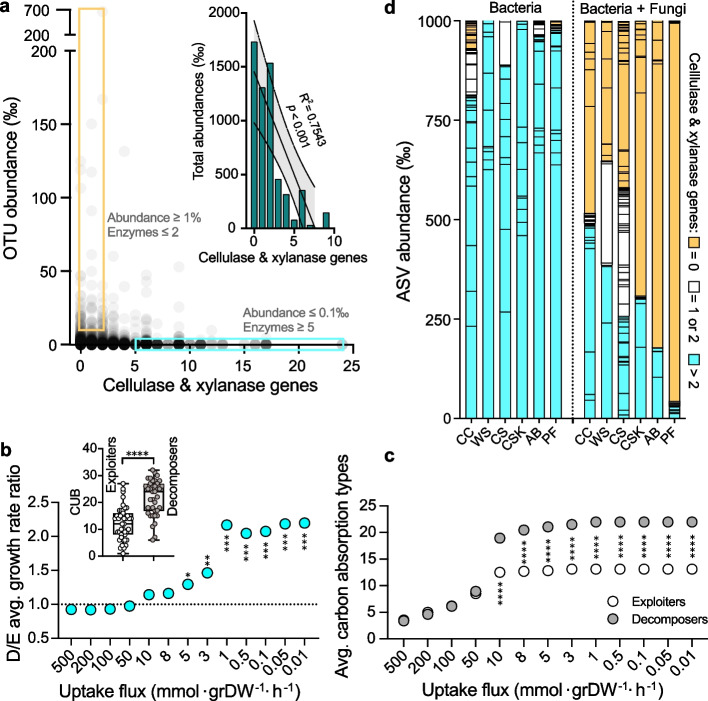
Exploitative dominance analysis of bacterial evolutionary communities. **a** The correlation between the abundance of bacterial OTUs and the cellulase and xylanase gene numbers in their matched genomes. Forty-three bacteria in the orange rectangle were exploiters (abundance ≥ 1% and gene count ≤ 2), and 40 bacteria in the turquoise rectangle were decomposers (abundance ≤ 0.1‰ and gene count ≥ 5). **b** FBA analysis-based average growth rate ratio of the decomposers (D) and exploiters (E) with a medium containing 35 plant residue-derived carbon sources. The inset shows the CUB variations. **c** The variation in average carbon absorption types across varying uptake fluxes between decomposers and exploiters. **d** The bacterial composition after 10 days of incubation by inoculating different synthetic microbiotas into six types of sterile plant residues (CC, WS, CS, CSK, AB, and PF). A synthetic microbiota (labeled Bacteria) contains 50 bacterial exploiters (cellulase and xylanase gene copy number = 0), 50 bacterial decomposers (gene copy number > 2) and 20 intermediate bacterial strains (gene copy number = 1 or 2), and another microbiota (labeled Bacteria + Fungi) contains the mentioned 120 bacterial strains and 50 fungal decomposers. Each grid represents a distinct amplicon sequence variant (ASV) unit, with different colors indicating the total number of cellulase and xylanase genes present in the ASV-matched bacterial genomes. Asterisks denote statistical significance: *, *p* < 0.05; **, *p* < 0.01; ***, *p* < 0.001; ****, *p* < 0.0001

Fungal decomposers dominated plant residue decomposition, while exploiters dominated the bacterial community. To address why the exploiters rather than the decomposers dominated the bacterial community, we first correlated the abundance of bacterial OTUs with the cellulase and xylanase genes in their OTU-matched bacterial genomes, and the results supported the dominance of exploiters at the abundance level (*R*^2^ = 0.75, *p* < 0.001) (Fig. 5a). Then, 43 exploiter genomes (enzymes ≤ 2, abundance ≥ 1%) and 40 decomposer genomes (enzymes ≥ 5, abundance ≤ 0.1‰) were selected for further analysis. To validate whether the dominant 43 exploiters would outperform the 40 decomposers under low carbon conditions, FBA was conducted to utilize the 35 carbon sources under different uptake fluxes. Surprisingly, the bacterial decomposers exhibited greater growth rates than bacterial exploiters (Fig. 5b, *p* < 0.05) under low carbon conditions (uptake flux ≤ 5 mmol·grDW^−1^·h^−1^) due to their significantly higher CUB values (22.0 vs. 12.1, Fig. 5b, *p* < 0.001), conferring decomposers with wide-ranging carbon uptake (Fig. 5c, *p* < 0.0001). This simulation was conflicted with the absolute advantage of exploiters observed in the evolutionary solid-phase subculturing. We speculated that the presence of fungal decomposers may switch the carbon acquisition strategy of bacterial communities. When fungal decomposers were absent, bacterial decomposers were the donor of carbon sources; they decomposed the plant residues and then utilized these carbons preferentially, thus dominating the community. However, when fungal decomposers were present, a higher concentration of available carbon sources were advantages for bacterial exploiters to achieve a greater growth rate than bacterial decomposers.

Thereafter, 50 fungal decomposers (Additional file 3: Fig. S6) and 120 bacterial strains (Additional file 6), including 50 bacterial exploiters (cellulase and xylanase gene copy number = 0), 50 bacterial decomposers (gene copy number > 2), and 20 intermediate bacterial strains (gene copy number = 1 or 2), were isolated from the 14th generation of solid-phase subculturing. A synthetic microbiota containing 120 bacterial strains and another microbiota containing 120 bacterial and 50 fungal strains were constructed and inoculated into the six types of sterile plant residues, respectively. After a 10-day fermentation period, DNAs were extracted for bacterial amplicon sequencing. The results showed that the bacterial decomposers dominate the bacterial community without the fungal decomposers (Fig. 5d, turquoise; Additional file 7), whereas the bacterial exploiters dominate the bacterial community with the presence of fungal decomposers (Fig. 5d, orange; Additional file 7). These results confirmed that fungal decomposers may have reversed the carbon acquisition strategy from a bacterial decomposer-dominated decomposition and utilization strategy to a fungal decomposer-dominated decomposition and bacterial exploiter-dominated utilization strategy.

## Discussion

Plant residue decomposition is ecologically crucial, and the driving factors behind the intricate interactions between fungi and bacteria in this process are generally unclear. This study corroborated our hypothesis that the residue complexity triggers divergent evolutionary trajectories for fungi and bacteria and is positively correlated with the contribution ratio of fungi/bacteria to decomposition. Remarkably, regardless of complexity variations, bacterial communities always predominantly act as exploiters, while fungal communities consistently serve as decomposers. In this context, bacteria adopted a more proactive exploitation strategy across the population, curtailing degrading enzymes and fully activating and controlling the transcription of OH and PBH genes to exploit and utilize the degradation products of fungi. Overall, the complexity of plant residues is the core driver for the intricate interactions between fungi and bacteria in decomposition, and the prevailing exploitation of fungi by bacteria remains constant, even in environments of low residue complexity.

To understand the intrinsic interaction mechanisms governing the prevalence of bacterial exploiters in the presence of fungi, the “exploiter-cooperator” framework (“exploiter-decomposer” herein) was introduced [27]. This theory suggests that decomposers ensure preferential access to public goods via strategies such as “public goods privatization” [30, 31] and “spatial structuring and limited mixed” [32]. Clearly, in plant residue decomposition, extracellular degrading enzymes are public goods. When bacteria exist alone, this priority also works at the bacterial community level, maintaining bacterial decomposer dominance, which has also been confirmed in the context of polysaccharide chitin decomposition by bacteria [33]. The presence of fungi in this study, however, disrupts this dynamic and conversely facilitates the bacterial exploiters. We propose that the extensive spread and enzyme secretion capabilities of fungal hyphae may fill the environment with public goods, thus challenging the priority of bacterial decomposers and reducing the reliance of bacterial exploiters on them. We noted that fungi facilitate rapid bacterial extension via a “hyphal highway” (Additional file 8). This raises questions regarding whether this hyphal interface creates habitats that are more conducive to bacterial survival, or whether this mode of transport exhibits evolutionary or functional selection, thus favoring bacterial exploiters. Our further findings corroborated this exploitative and dependent relationship of bacteria on fungi, that is, when bacteria were present alone, their extension rate (0.031 ± 0.008 mm·h^−1^) and biomass (bacterial DNA: 0.51 μg·g^−1^·FW) were highly significantly lower than those observed in coexistence with fungi (extension rate: 1.87 ± 0.22 mm·h^−1^; total DNA: 33.12 μg·g^−1^·FW with a 16S rDNA to ITS ratio of approximately 1:1, Additional file 3: Fig. S7). Additionally, the inherent high-secretory nature of bacterial decomposers signifies a substantial resource burden on their unicellular form, a challenge that may be exacerbated by ecological niche overlap with fungal decomposers. This raises the question: does such overlap induced competition between bacterial and fungal decomposers? Notably, bacterial antagonism plays a critical role in shaping community coexistence [34], and the prevalent antagonism between bacteria and fungi [26] may drive the evolutionary dominance of bacterial exploiters. To this end, we further analyzed the distribution and expression of secondary metabolite biosynthetic gene clusters (smBGCs) within fungal and bacterial communities in plant residue decomposition. A total of 2664 fungal smBGCs (76 OTU-matched genomes) and 3172 bacterial smBGCs (421 OTU-matched genomes) were identified, with significant differences in average smBGC numbers and group distributions between fungi and bacteria (Additional file 3: Fig. S8a). These findings provide a basis for understanding community dynamics. In metatranscriptomes, approximately 57.6% of smBGCs were expressed (fungi: 51.5%, bacteria: 62.7%). Bacterial smBGC expression exhibits a significant negative correlation with residue complexity (*R*^2^ = 0.68, *p* < 0.0001, Additional file 3: Fig. S8b), corroborating the ecological concept that microbes engage in stronger competition under nutrient-rich conditions (low-complexity residues) and weaker competition under nutrient-poor conditions (high-complexity residues) [35–38]. In contrast, fungal BGC expression was positively correlated with residue complexity (*R*^2^ = 0.45, *p* < 0.01, Additional file 3: Fig. S8b), where nutrient scarcity drove stronger competition, challenging the conventional ecological theories and necessitating further exploration. However, this intensification of fungal competition supports the observed increase in fungal richness and sharp decline in bacterial richness with increasing residue complexity, offering insights into cross-kingdom antagonistic interactions. The sharp decline in bacterial exploiter smBGC expression reflects a strategic compromise in competition, as they rely on fungal decomposers for survival. Furthermore, we focused on bacterial exploiters, whose smBGC expression was also negatively correlated with residue complexity (*R*^2^ = 0.76, *p* < 0.0001, Additional file 3: Fig. S8b). Bacterial exploiters dominated communities, and thus their smBGC responses shaped the whole community dynamics. However, bacterial decomposers, like fungi, exhibit a significant positive correlation between smBGC expression and residue complexity (*R*^2^ = 0.47, *p* < 0.01, Additional file 3: Fig. S8b). This independent smBGC response in bacterial decomposers clearly reflects autonomous adaptation and regulation, as no significant differences were observed in genomic smBGC distribution between bacterial decomposers and exploiters (Additional file 3: Fig. S8a). Therefore, it can be inferred that ecological niches determine interaction strategies. Bacterial and fungal decomposers share the same ecological niche in plant residue decomposition, which leads to similar competitive strategies with stronger competition under high-complexity residues. This suggests their potential direct antagonism, possibly explained the consistently low proportion of bacterial decomposers. Although a relatively small proportion of expressed smBGCs were corresponded to known product clusters (fungi: 24.5%, bacteria: 46.0%), we observed specific BGC expression profiles for fungal decomposers (PKSI), bacterial decomposers (PKSother and RiPPs), and bacterial exploiters (Terpene) (Additional file 3: Fig. S8c), in which high-expression products, including asperthecin, oosporein, oxyjavanicin, loseolamycin, difficidin, lankacidin, paeninodin, berninamycin and misaugamycin, exhibit significant antimicrobial activities. Overall, the trade-off in competitive intensity between exploiters and decomposers, driven by the nutrient gradient of plant residue complexity, showcases the art of survival, offering additional support for the dynamic evolution of microbial communities.

Undoubtedly, uncontrolled exploitative behavior can destabilize systems and potentially eradicate decomposers. This prompts an inquiry into the strategies by which fungal decomposers gain preferential access to the public goods of degrading enzymes. Fungal hyphae, with their rapid extension and tip-specific secretion [39], maintained a bacteria-free tip during plant residue decomposition (Additional file 3: Fig. S9). This highlights fungi’s inherent proficiency in carbon acquisition via rapid extension, secretion, and efficient residue processing. Given the quick enzymatic release of easily degradable substrates from plant residues (Additional file 3: Fig. S9), the advantages of the fungal approach become evident. This mechanism, analogous to “spatial structuring and limited mixing”, is logically and observationally coherent, but its proportional impact still awaits quantification. Moreover, as multicellular eukaryotes, fungi have advantages in environmental adaptability, resource allocation, and cellular collaboration compared to bacteria [40]. Beyond these punitive interactions, we cannot exclude the possibility that bacterial exploiters and fungal decomposers might establish cooperative relationships. For instance, plant residues exhibit a high C/N ratio, and numerous reports have suggested that bacteria might provide N sources to fungi through nitrogen fixation [8, 41]. This prompts our interest in probing the cooperative C and N dynamics between fungi and bacteria during plant residue decomposition as well as other potential cross-feeding interactions. Such interactions could enhance the stability of bacterial exploitation of fungi. Additionally, as we observed, the bacterial contribution to decomposition was enhanced under low residue complexity. Logically, this could be attributed to the decomposability and sustained decomposition effects of low-complexity residues, providing sufficient carbon sources and supporting the micro-environment for bacterial decomposers. This phenomenon aligns with a report that highlights the prominent contribution of low-abundance bacteria to straw decomposition [16]. Understandably, this can be interpreted as a compensatory effect mediated by low-proportion bacterial decomposers against the backdrop of bacterial exploitation of fungi. Overall, the fungal and bacterial decomposition system might be grounded in a game model based on stable exploitation and dynamic cooperation.

Despite efforts to elucidate the functional roles of fungi and bacteria and their interactions in plant residue decomposition, natural environments are likely to be more diverse and complex. For instance, we did not retain the initial mixed microbial consortia due to their complexity, instability, and the conditioning imprints. However, such analysis of natural decomposing communities could potentially yield valuable insights. Moreover, temperature is a key determinant of plant residue turnover rates and the efficiency of organic matter accumulation in soil [42]. Adaptation differences between fungi and bacteria to extreme temperatures possibly influence their contributions to decomposition. Fungi are better adapted to drought [43], while higher moisture levels promote bacterial extension. Additionally, soil salinity regulates fungal decomposition efficiency via hyperosmotic-response pathway [44], and pH not only shapes bacterial diversity [45] but also significantly affects fungal ability of plant residue decomposition [46]. Incorporating these factors such as temperature, pH, moisture, and salinity will enable a more comprehensive understanding of the microbial mechanisms driving plant residue decomposition.

## Conclusions

This study reveals a remarkable interaction between fungi and bacteria in plant residue decomposition, a crucial ecological process in soil carbon turnover. Previously, both fungi and bacteria were known to contribute, however, their precise roles remained ambiguous. Now, we see a distinct interplay: fungi consistently dominate decomposers, regardless of plant residue complexity, while bacteria strategically utilize fungal degradation products, thereby consistently acting as exploiters. This refined view of their “decomposer-exploiter” relationship significantly enhances our grasp of the microbial mechanisms that drive organic carbon cycling.

## Methods

### Experimental setup of successive solid-phase subculturing

To evolve stable microbial communities capable of efficiently decomposing diverse plant residues, we collected 127 samples of potential plant residue-decomposing microorganisms from various natural habitats, processed them into a homogenized mixture, and utilized this as the initial microbial consortia. These samples were obtained from artificial composting systems (straw compost and mixed agricultural waste compost; 30–60 °C, 40–60% moisture), primary forests in Inner Mongolia, China (litter and humic horizons; 5–20 °C, 45–70% relative humidity; coordinates: 121.45°E-52.37°N, 122.50°E-51.44°N, 121.41°E-48.42°N, 123.35°E-49.54°N, and 120.46°E-49.06°N), Zijin Mountain in Nanjing, China (litter and humic horizons; 25–35 °C, 40–70% relative humidity; coordinates: 118.48°E-32.02°N, and 118.50°E-32.05°N), and typical grasslands in Inner Mongolia, China (surface soil; 10–20 °C, 25–45% relative humidity; coordinates: 120.90°E-42.85°N, 118.87°E-42.53°N, 116.67°E-43.55°N, and 116.16°E, 43.20°N). Six types of plant residues with a gradient variation in composition, including corn cob, wheat straw, corn stalk, cotton stalk, apple branch, and palm fiber, were used for solid-phase subculturing. The complexity of plant residue was defined here by the proportion of lignin and cellulose, which is key to resisting microbial degradation [18]. Before inoculation, the plant residues were pulverized and passed through a 40-mesh sieve and then transferred (2 cm-thick) into a rectangular container (17.5 cm × 9.5 cm) with a moisture content of 120% (w/w) adjusted using liquid MM medium (5 g·L^−1^ (NH_4_)_2_SO_4_, 15 g·L^−1^ KH_2_PO_4_, 0.6 g·L^−1^ MgSO_4_·7H_2_O, 1 g·L^−1^ CaCl_2_, 1.6 mg·L^−1^ MnSO_4_·H_2_O, 1.4 mg·L^−1^ ZnSO_4_·7H_2_O, 5 mg·L^−1^ CoCl_2_·H_2_O), followed by sterilization. The homogenized environmental inoculum was inoculated at both ends of the container (Fig. 1a) and incubated at 28 °C for 15 days. Then, each incubated plant residue was homogenized thoroughly as a new inoculum to inoculate the same fresh plant residue container for the next generation of incubation. A total of 14 generations were conducted. The Van Soest analytical procedure [47] was applied to detect the contents of cellulose, hemicellulose, and lignin in plant residues before and after the incubation period of each generation. Each sample was detected with three biological replicates.

### DNA extraction and amplicon sequencing analysis

Three replicate samples of each type of plant residue were collected on the 7th day of fermentation in the 14th generation of solid-stage fermentation. Total DNA was extracted from a 0.25 g sample using the QIAGEN DNeasy PowerSoil Pro Kit (Ref: 12,888–100, Germany). The DNA quality was assessed using a NanoDrop ND-2000 spectrophotometer (Thermo Scientific, USA). The V4-V5 regions of the bacterial 16S rRNA gene (341F, 5′-CCTAYGGGRBGCASCAG-3′; 806R, 5′-GGACTACNNGGGTATCTAAT-3′) and the fungal internal transcribed spacer (ITS) region (ITS1F, 5′-CTTGGTCATTTAGAGGAAGTAA-3′; ITS2R, 5′-GCTGCGTTCTTCATCGATGC) were amplified to assess the bacterial and fungal communities, respectively. The PCR amplicons were paired-end (2 × 250) sequenced on an Illumina MiSeq platform (BIOZERON Co., Ltd., Shanghai, China), and the sequencing data were processed using the UPARSE pipeline (version 11.0, http://drive5.com/uparse/). After removing adapters and primers, the sequences were subjected to quality control (quality score 20), and the short (< 50 bp) and unpaired reads were trimmed. The remaining sequences were assembled, followed by removing the singletons and chimeric sequences, and then categorized into operational taxonomic units (OTUs) at 97% similarity. The DNA sequences were deposited in the National Center for Biotechnology Information (NCBI) under accession number PRJNA1004139 [48].

The OTU table was rarefied to 28,913 and 29,288 reads per sample for bacterial and fungal communities, respectively. The relative abundance of a phylogenetic group (OTU) was defined as the number of reads affiliated with that group divided by the total number of reads per sample. The nearest taxon index (NTI) analysis was performed using the picante R package (v1.8.2, https://github.com/skembel/picante). NTI values > 2 indicate phylogenetic clustering, whereas NTI values < − 2 indicate phylogenetic overdispersion within a community [49]. The richness index calculation and Bray–Curtis distance-based principal coordinate analysis (PCoA) of the bacterial and fungal communities were performed using the rarefied OTU table by the vegan R package (v2.6–4, https://github.com/vegandevs/vegan).

### Bacterial and fungal genome analysis

A total of 16,063 bacterial and 1503 fungal non-redundant genomes were obtained from the NCBI GenBank and JGI databases. In the incubation samples, 2190 bacterial and 309 fungal OTUs were detected. Following the methods of Aleksej et al. [50] and Daniel et al. [51], the genomes of the detected bacterial and fungal OTUs were matched and selected from the 16,063 bacterial and 1501 fungal genomes, respectively. Briefly, we initially applied a stringent sequence similarity cutoff (≥ 97% sequence identity, ≥ 95% query sequence overlap) and achieved genomes for 53.2% (1164/2190) and 30.7% (95/309) of the bacterial and fungal OTUs, respectively. These OTUs represented 85.6% and 92.1% of the bacterial and fungal sequences, respectively. For the remaining OTUs, we adjusted the sequence identity to ≥ 95% and achieved 374 and 31 more genomes for the bacterial and fungal OTUs, respectively. Overall, we achieved genomes for 70.2% (1538/2190) and 40.8% (126/309) of the bacterial and fungal OTUs, respectively. These OTUs represented up to 92.8% and 93.5% of the bacterial and fungal sequences, respectively. Notably, an OTU might have been matched to more than one genome; when this occurred, we retained only one genome with the highest similarity. Additionally, a given genome could be mapped to multiple OTUs.

The CAZyme annotations for all genomes were achieved using the HMMER 3.0 tool (http://www.hmmer.org) to search the dbCAN HMM database (release 11.0, http://www.bcb.unl.edu/dbCAN/). The subsequent functional classification of CAZyme families was performed using Bash scripts with cellulase families including GH5-1, GH5-2, GH5-22, GH5-25, GH5-26, GH5-37, GH5-38, GH5-4, GH5-46, GH5-5, GH5-52, GH5-53, GH5-55, GH6, GH7, GH8, GH9, GH12, GH44, GH45, GH48, GH74, GH124, GH131, AA9, AA10, AA3-1, AA15, and AA16; xylanase families including GH5-21, GH5-34, GH5-35, GH10, GH11, GH30-7, GH30-8, AA14, GH43-17, and GH43-7; ligninolytic enzyme families including AA1-1, AA1-2, AA1-3, and AA2; and the families of OH and PBH comprising GH1, GH2, GH3, GH116, GH39, GH40, GH43-1, GH43-10, GH43-11, GH43-12, GH43-14, GH43-16, GH43-18, GH43-19, GH43-2, GH43-20, GH43-21, GH43-22, GH43-23, GH43-26, GH43-27, GH43-29, GH43-30, GH43-32, GH43-33, GH43-34, GH43-35, GH43-36, GH43-37, GH43-4, GH43-5, GH43-9, GH5-43, GH5-45, CE1, CE2, CE3, CE4, CE5, CE6, CE7, CE12, CE16, GH5-9, GH95, GH62, GH67, GH52, GH54, and GH120. For the fungal genomes lacking annotations, we performed protein annotation using Augustus v2.5.5 (https://bioinf.uni-greifswald.de/augustus/).

To estimate the plant residue-degrading potential of each bacterium and fungus, we constructed a multi-metric weighted ranking calculation to generate a plant residue decomposition index (PDI) for each genome based on the quantity and diversity of the residue decomposition-related CAZymes, which, along with multi-enzyme synergy, was assumed to correlate closely with microbial capability in decomposing plant residues. The PDI value of individual microbes was calculated by the Eq. 1:

1 $${PDI}_{i}=\frac{{D}_{ci}}{{T}_{c}}\times 30\%+\frac{{D}_{xi}}{{T}_{x}}\times 30\%+\frac{{D}_{li}}{{T}_{l}}\times 5\%+\frac{{D}_{opi}}{{T}_{op}}\times 10\%+\frac{{D}_{gci}}{{T}_{gc}}\times 10\%+\frac{{D}_{fi}}{{T}_{f}}\times 10\%+\frac{{D}_{di}}{{T}_{d}}\times 5\%$$

where *PDI*_*i*_ is the plant residue-degrading potential of microbe *i*. The terms *D*_*ci*_, *D*_*xi*_, *D*_*li*_, *D*_*opi*_, *D*_*gci*_, *D*_*fi*_, and *D*_*di*_ represent the gene quantities of cellulase, xylanase, ligninolytic enzyme, OH and PBH, CAZymes excluding glycosyltransferases (GTs), families of cellulase, xylanase and OH and PBH, and families of CAZyme excluding GTs, respectively, in the genome of microbe *i*. The *T*_*c*_, *T*_*x*_, *T*_*l*_, *T*_*op*_, *T*_*gc*_, *T*_*f*_ and *T*_*d*_ are the sums of *D*_*ci*_, *D*_*xi*_, *D*_*li*_, *D*_*opi*_, *D*_*gci*_, *D*_*fi*_, and *D*_*di*_ of all microbes. The weights of these seven parameters are empirically assigned to 30%, 30%, 5%, 10%, 10%, 10%, and 5%, respectively. Here, the highest weights were assigned for cellulase and xylanase because they contribute to decomposition of the two most abundant polysaccharides in plant residues. However, the lowest weight was assigned for ligninolytic enzymes due to their inefficiency to release carbon sources.

### Metatranscriptomic sequence analysis

Total RNA was extracted from the same samples for amplicon sequencing, using TRIzol Reagent (Invitrogen, USA). Then, high-quality RNA samples (OD_260/280_ = 1.8 ~ 2.2, OD_260/230_ ≥ 2.0, RIN ≥ 6.5, 28S:18S ≥ 1.0) were determined using a 2100 Bioanalyzer (Agilent Technologies, USA) and quantified using a NanoDrop ND-2000 spectrophotometer (Thermo Scientific, USA). Metatranscriptome libraries were constructed following the Illumina’s TruSeq stranded total RNA sample preparation kit (San Diego, CA), culminating in paired-end sequencing (2 × 150 bp) on an Illumina HiSeq 2500 platform (BIOZERON Co., Ltd., Shanghai, China). The raw data can be accessed in the NCBI database under accession number PRJNA1004139 [48].

The raw sequencing data were subjected to a quality check via FastQC v0.12.1 and to subsequent cleaning using Trim_galore v0.6.9 (parameters: –quality 25 –stringency 1 –length 30 –paired). The clean data of six types of plant residue were then individually subjected to de novo assembly through Trinity v2.15.0. Afterward, sequences were annotated into genes and encoding protein sequences (≥ 80 AA) via TransDecoder v5.7.0, adhering to ORF retention criteria that included homology searches to Uniref90 and Pfam databases. For each gene, taxonomic assignment was first performed through protein-space sequence similarities with the UniRef90 database, computed by DIAMOND v2.1.3 with -very-sensitive mode. With the output, the matched proteins in the database having ≥ 95% bitscore of the highest match were retained, and their TaxIDs were parsed using TaxonKit v0.14.2. Finally, genes were classified as fungal or bacterial based on whether over 50% of their matched entries originated from fungi or bacteria. The transcript abundance, represented by transcripts per million transcripts (TPM), was quantified using the internal Trinity script of “align_and_estimate_abundance.pl.” For comparative analyses across samples, protein sequences were clustered using CD-HIT v4.8.1 with cutoffs of 40% sequence identity and 80% alignment coverage (parameters: -c 0.4 -n 2 -aS 0.8). Each cluster represented a unique homologous protein functional group (HPFG), serving to integrate the genes across all the samples. TPM abundances of all HPFGs across different samples were aggregated using bash commands and normalized via the Trimmed Mean of M-values (TMM) method, facilitated by the Trinity script of “abundance_estimates_to_matrix.pl.”

The annotated CAZyme genes from the metatranscriptome were aligned with the achieved genomes of the detected bacterial and fungal OTUs via BlastP in BLAST + 2.14.1 using a stringent criterion of ≥ 70% sequence identity and ≥ 70% alignment coverage. To compare the transcriptional differences of the enzyme groups (cellulases, xylanases, ligninolytic enzymes, and OHs and PBHs) between fungi and bacteria across samples, the TPM values of all genes within each group were summed to calculate the expression proportion.

### Genome-scale metabolic model construction and flux balance analysis

The genome-scale metabolic models (GMMs) were constructed using the CarveMe v1.5.0 software, and each metabolic model was then subjected to gap-filling with glucose-containing MM medium. Afterward, all exchange reactions were extracted from these GMMs to filter the carbon sources that could theoretically be the decomposition products of plant residue. A total of 35 carbon sources were selected (Additional file 5). Then, to calculate the carbon utilization breadth (CUB) of each bacterium, flux balance analysis (FBA) was performed by the COBRA Toolbox v3.0 on MM medium, in which the carbon source was replaced by one of the 35 carbon sources for each reaction. Here, we applied an unrestricted uptake flux (default maximum flux of 1000 mmol·grDW^−1^·h^−1^) to optimize bacterial growth. Therefore, a growth rate above zero and CO_2_ release represented the ability to utilize the specific carbon source. The total number of carbon sources that a bacterium can utilize indicates its CUB.

To simulate the bacterial growth features during plant residue decomposition, the FBA of each GMM was conducted using MM medium containing all 35 carbon sources at distinct uptake flux thresholds (0.01, 0.05, 0.1, 0.5, 1, 3, 5, 8, 10, 50, 100, 200, 500, and 1000 mmol·grDW^−1^·h^−1^). The average number of absorbed carbon sources (ANAC) on each uptake flux level was calculated by Eq. 2. The carbon secretion types (CST) were calculated using Eq. 3, indicating the number of bacterial secreted carbon sources (excluding CO_2_). Carbon secretion efficiency (CSE) was calculated as the ratio of secreted carbon sources (excluding CO_2_) to total absorbed carbon sources (Eq. 4). In the simulation process, to simplify the system, we did not consider the constraints of spatial limitations and nutrient consumption. Hence, community-related features and metrics could be statistically obtained from individual bacteria data. The community CSE (CCSE) was calculated as the growth-rate-weighted average of CSEs of individual GMM within the community (Eq. 5). The variability of the community growth rate (CGRV) with uptake flux was calculated using Eq. 6.

2 $$ANAC\;=\;\frac{\sum_{i=1}^jN_i}j$$

3 $$\text{CST}={\bigcup }_{i=1}^{j}{M}_{i}$$

4 $${CSE}_{i}=\frac{{\sum }_{m=1}^{{M}_{i}}{Secretion flux}_{m}\times {C}_{m}}{{\sum }_{n=1}^{{N}_{i}}U{ptake flux}_{n}\times {C}_{n}}$$

5 $$\text{CCSE}={\sum }_{i=1}^{j}(\frac{{Growth rate}_{i}}{{\sum }_{k=1}^{j}{Growth rate}_{k}}\times {CSE}_{i})$$

6 $$CGRV=\frac{\left({\sum }_{i}^{j}{Growth rate}_{i,flux=a}-{\sum }_{i}^{j}{Growth rate}_{i,flux=b}\right)/j}{a-b}$$

where *N*_*i*_ and *M*_*i*_ are the number of absorbed and secreted carbon sources, respectively, by microbe *i*. *j* is the number of GMMs used in this simulation. *n* and *m* represent each absorbed and secreted carbon source, respectively, by microbe *i*. C signifies the count of carbon atoms. *k* serves as a dummy variable for summing the growth rates of all GMMs in the community.

When simulating cross-feeding within a bacterial community, it is essential to consider the variation in CCSE in response to different uptake fluxes, thereby resulting in changes in the cross-feeding effect. First, FBA on 421 bacterial GMMs was performed at a specified carbon uptake flux using MM medium containing 35 plant-residue-derived carbon sources. This yielded total *X* distinct carbon-containing secretions (excluding CO_2_), each potentially co-secreted by *Y* bacterial GMMs, with their secretion fluxes being cumulatively added. Secretions below 0.01 mmol·grDW^−1^·h^−1^ flux were not considered. Subsequently, a rule was set to ensure that the total C flux of carbon-containing secretions did not exceed the product of the total C uptake flux and the corresponding CCSE as Eq. 7.

7 $$\beta \times {\sum }_{p=1}^{X}({\sum }_{q=1}^{{Y}_{p}}{Secretion flux}_{q}\times {C}_{p})\le CCSE\times {\sum }_{t=1}^{S}{Uptake flux}_{t}\times {C}_{t}$$

where *p* represents each secretion, *Y*_*p*_ signifies the number of GMMs producing secretion *p*, *q* stands for each repeated secretion of *p*, *S* is the total 35 carbon sources uptake, *t* represents each uptake carbon source, and C signifies the count of carbon atoms.

Subsequently, by integrating carbon-containing secretions following the rule of Eq. E7 and the 35 carbon sources as inputs, a refined FBA was executed on 421 bacterial GMMs under various uptake flux parameters. This represented a community growth simulation that comprehensively incorporated the interspecies bacterial cross-feeding effects. The relative divergence (RD) induced by cross-feeding on the bacterial community’s average growth rate (G) can be calculated as follows (Eq. 8):

8 $$RD=\frac{{G}_{cross-feeding}-{G}_{non}}{\left({G}_{cross-feeding}+{G}_{non}\right)/2}$$

### Synthetic microbiota construction and incubation

The samples collected from the 14th incubation generation were used for isolation of bacteria and fungi. The bacterial and fungal isolates were collected from TSB and PDA agar plates, respectively. Bacteria were classified using 16S rRNA. Their matched genomes were obtained as described in the bacterial and fungal genome analysis section. Fifty fungal decomposers (Additional file 3: Fig. S5) and 120 bacterial strains (Additional file 6) were isolated, including 50 bacterial exploiters (cellulase and xylanase gene copy number = 0), 50 bacterial decomposers (gene copy number > 2) and 20 intermediate bacterial strains (gene copy number = 1 or 2). We constructed two synthetic microbiota, one containing 120 bacterial strains and another containing 120 bacterial and 50 fungal strains. To generate a synthetic microbiota, the strains were cultured separately and then mixed with an equal number of cells or spore counts. The incubation container of six types of sterile plant residues was established as described in the experimental setup of successive solid-phase subculturing section. DNA was extracted as described in the section on DNA extraction and amplicon sequencing analysis and then quantified precisely using a Qubit 4.0 fluorometer (Thermo Fisher Scientific, USA). For qPCR analysis, DNA samples were adjusted to a final concentration of 5 ng·μL^−1^ and were then subjected to analysis using a 7500 Fast Real-Time PCR System (Applied Biosystems, USA) with the SYBR premix Ex Taq kit (TaKaRa, China). This qPCR analysis was used for the identification and differentiation of fungal and bacterial DNA presence using primers targeting bacterial 16S rDNA (314F/805R) and fungal ITS (ITS1/ITS2). Through direct observation of hyphal extension, microscopic examination of bacteria, DNA-based biomass detection, and bacterial qPCR verification, the extension positions of fungi and bacteria were identified, enabling the calculation of their extension rates under different conditions. Specifically, fungal hyphal extension tips (1–2 cm) in the mixed cultures were selectively processed to distinguish the presence or absence of bacteria by qPCR analysis. At the end of the 10-day fermentation period, substrates located at 1 cm, 6 cm, 11 cm, and 16 cm distances from the inoculation point were sampled and utilized for DNA extraction to assess bacterial and fungal biomass. Concurrently, plant residue samples within the range of microbial extension were homogenized separately and then subjected to an additional round of DNA extraction for bacterial amplicon sequencing. The raw data can be accessed in the NCBI database under accession number PRJNA1004139 [48]. Extracellular enzymes within the samples were collected through a series of steps, including sterile water dissolution, agitation, centrifugation for supernatant, and 80% ammonium sulfate precipitation. Equal amounts (2% (w/v)) of different plant residues were mixed with an equal quantity of enzymes and subjected to reactions at pH 5.0, 100 rpm, and 28 °C. Changes in released reducing sugars were detected using the DNS method. Each analysis above was performed with three replicates.

### Secondary metabolite biosynthetic gene cluster analysis

Secondary metabolite biosynthetic gene clusters (smBGCs) from OTU-matched 76 fungal and 421 bacterial genomes were identified and annotated using ANTISMASH 7.1.0. The protein products of smBGCs were searched against the metatranscriptomes using DIAMOND v2.1.3 in ultra-sensitive mode, with stringent criteria of ≥ 70% sequence identity and ≥ 70% alignment coverage. For each matched metatranscriptomic protein, only the most similar smBGC protein was retained. When a single smBGC protein matched multiple metatranscriptomic proteins, the TPM values of the corresponding genes were summed to represent the smBGC protein’s expression level. The expression of a smBGC was calculated as the average expression of all its matched proteins. Total smBGC expression for fungal or bacterial taxa was determined by summing the TPM values of all smBGCs within each taxonomic group.

## Supplementary Information

Additional file 1. The CAZyme annotation of 16,063 bacterial and 1501 fungal genomes.

Additional file 2. The OTU tables of the evolved bacterial and fungal communities.

Additional file 3: Supplementary figures. Fig. S1. Statistical analysis of metatranscriptomic data. Fig. S2. PCoA analysis of bacterial and fungal non-redundant protein clusters. Fig. S3. Distribution of CAZymes in metatranscriptomes. Fig. S4. Enzymatic correlation with residue complexity in metatranscriptomic samples. Fig. S5. CUB comparison between the 421 evolved bacterial GMMs and random bacterial GMMs from the database. Fig. S6. PDA plate images of 50 fungal decomposers. Fig. S7. DNA-related biomass evaluation in the solid-state fermentation of the synthetic microbiota. Fig. S8. Analysis of secondary metabolite biosynthetic gene clusters (smBGCs) in fungal and bacterial communities of plant residue decomposition. Fig. S9. Bacteria-free confirmation of fungal hyphal tips and hydrolysis analysis.

Additional file 4. The annotated CAZymes in metatranscriptomes.

Additional file 5. Thirty-five plant residue-derived carbon sources and 71 secretions produced by 421 bacterial GMMs.

Additional file 6. Total 120 bacterial strains.

Additional file 7. The bacterial ASV tables for only bacteria and bacteria + fungi fermentations.

Additional file 8. Bacteria traversing fungal aerial mycelial highways. In the co-cultivation of fungi and bacteria during the plant residues decomposition, the effect of 1000× magnification under an optical microscope shows aerial hyphae of fungi, crisscrossing in the image. On them, directionally flowing, slightly greenish light points represent bacteria.

Additional file 9. Review history.

## Data Availability

The raw data generated in this study have been deposited in the National Center for Biotechnology Information BioProject database under accession number PRJNA1004139 [48].
