# Supplementary material for: A novel decomposer-exploiter interaction framework of plant residue microbial decomposition
Source: Genome Biol. 2025 Feb 3;26:20. doi: 10.1186/s13059-025-03486-w (PMC11792400; doi:10.1186/s13059-025-03486-w)
Supplement: Supplementary file 3 — Additional file 3: Supplementary figures. Fig. S1. Statistical analysis of metatranscriptomic data. Fig. S2. PCoA analysis of bacterial and fungal non-redundant protein clusters. Fig. S3. Distribution of CAZymes in metatranscriptomes. Fig. S4. Enzymatic correlation with residue complexity in metatranscriptomic samples. Fig. S5. CUB comparison between the 421 evolved bacterial GMMs and random bacterial GMMs from the database. Fig. S6. PDA plate images of 50 fungal decomposers. Fig. S7. DNA-related biomass evaluation in the solid-state fermentation of the synthetic microbiota. Fig. S8. Analysis of secondary metabolite biosynthetic gene clusters (smBGCs) in fungal and bacterial communities of plant residue decomposition. Fig. S9. Bacteria-free confirmation of fungal hyphal tips and hydrolysis analysis. [file 13059_2025_3486_MOESM3_ESM.pdf]

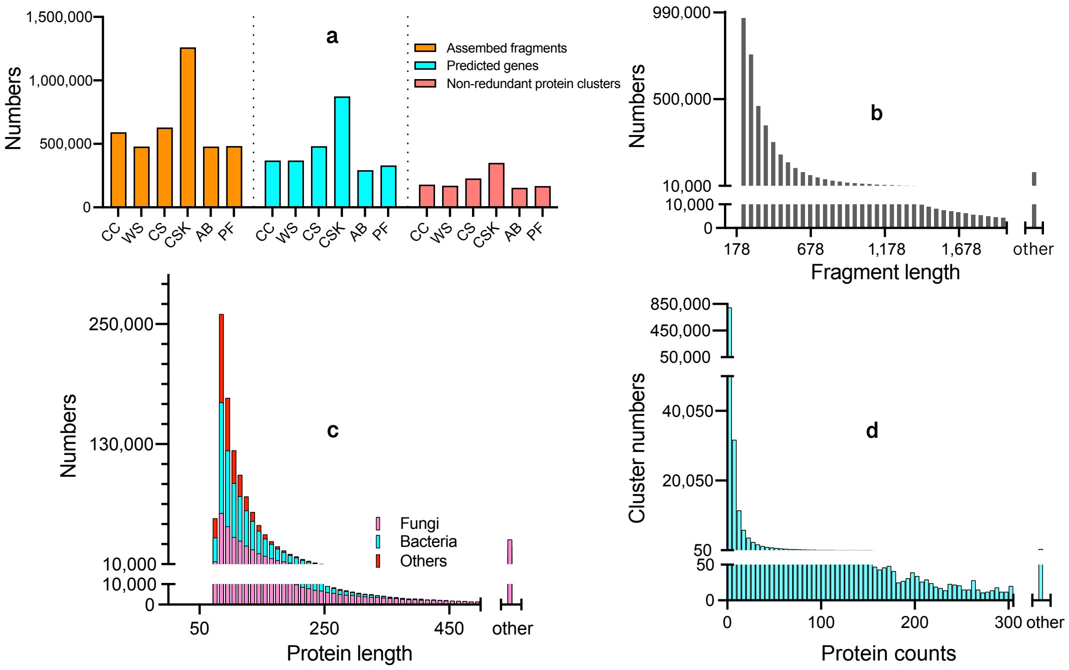

Fig. S1. Statistical analysis of metatranscriptomic data. Through sequencing, metatranscriptomes from distinct plant residues (CC, WS, CS, CSK, AB and PF) yielded corresponding assembly fragment numbers, predicted gene counts, and non-redundant protein clusters (cd-hit: 40% identity and 80% coverage, **see Methods**) (**a**). Density histograms illustrate the distributions of assembly fragment lengths (**b**), predicted protein lengths (**c**), and protein counts within non-redundant protein clusters (**d**) across all samples.

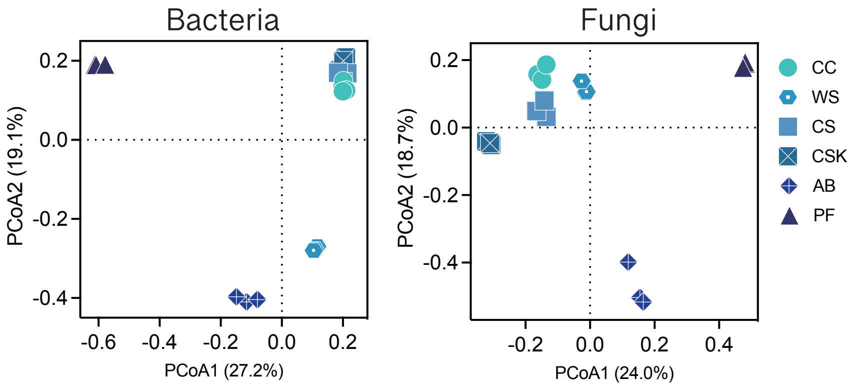

Fig. S2. PCoA analysis of bacterial and fungal non-redundant protein clusters. Metatranscriptomic analysis resulted in a total of 862,953 non-redundant protein clusters, in which 268,919 bacterial and 112,856 fungal protein-containing clusters were included. Thus, Bray–Curtis-based PCoA was performed using the transcriptional data of these bacterial (**a**) and fungal (**b**) clusters, respectively, to demonstrate their contributions to metatranscriptomic variation across various plant residues (CC, WS, CS, CSK, AB and PF).

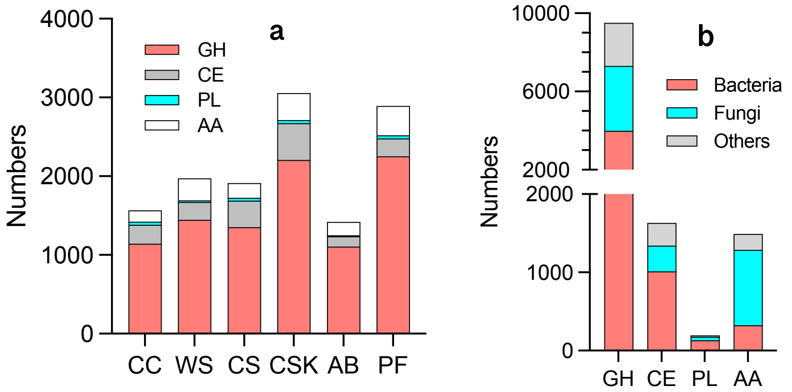

Fig. S3. Distribution of CAZymes in metatranscriptomes. A total of 12,813 CAZymes belonging to the GH (glycoside hydrolase), CE (carbohydrate esterase), PL (polysaccharide lyase) and AA (auxiliary activity) families were identified and represented as a histogram across different metatranscriptomic samples (CC, WS, CS, CSK, AB and PF) (**a**). Additionally, the distribution of fungal and bacterial protein counts within each CAZyme family is shown in (**b**).

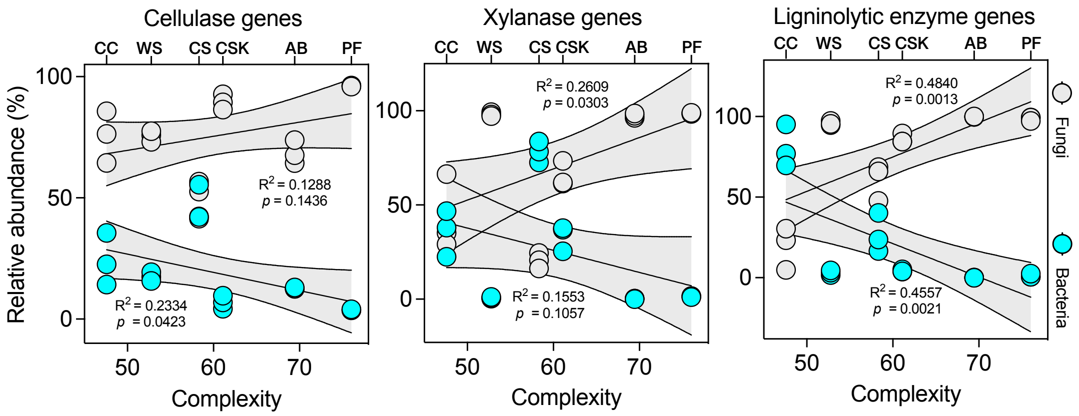

Fig. S4. Enzymatic correlation with residue complexity in metatranscriptomic samples. In the metatranscriptomes derived from six plant residues (CC, WS, CS, CSK, AB and PF), the individual abundances of bacterial and fungal cellulases, xylanases, and ligninolytic enzymes were calculated to examine their relationships with plant residue complexity.

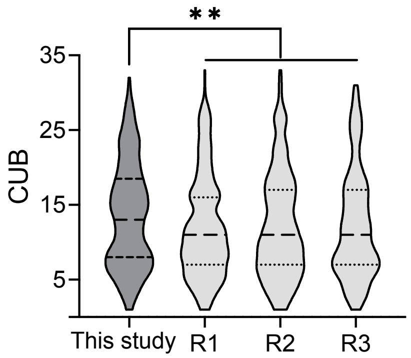

Fig. S5. CUB comparison between the 421 evolved bacterial GMMs and random bacterial GMMs from the database. The CUB distribution of the 421 evolved bacterial GMMs is shown as a violin plot, and was compared with three random bacterial communities (R1, R2 and R3) selected from the database of 16,063 non-redundant bacterial genomes. ** means $p < 0.01$.

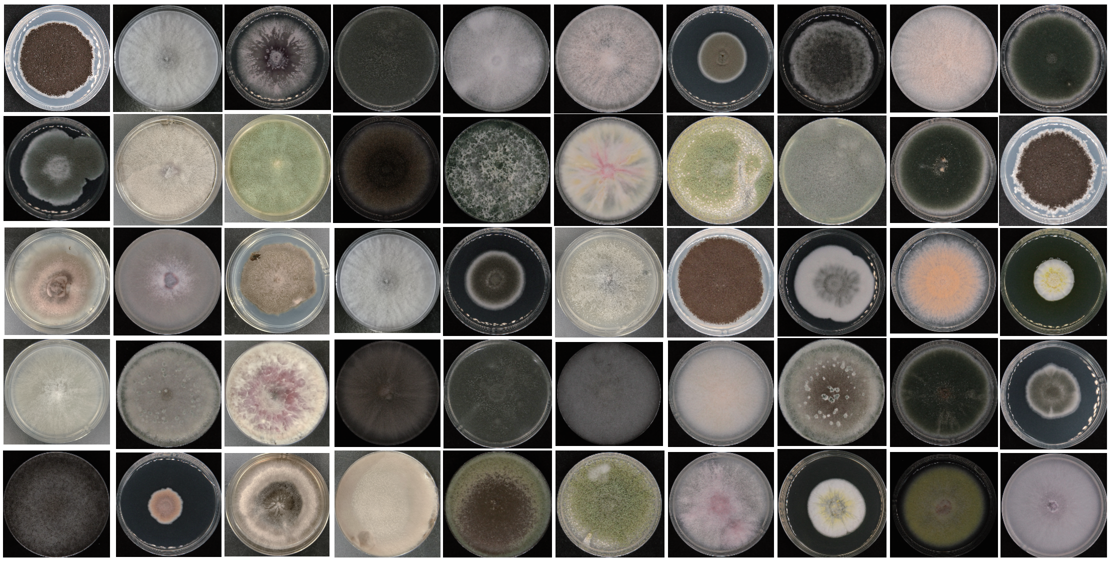

Fig. S6. PDA plate images of 50 fungal decomposers.

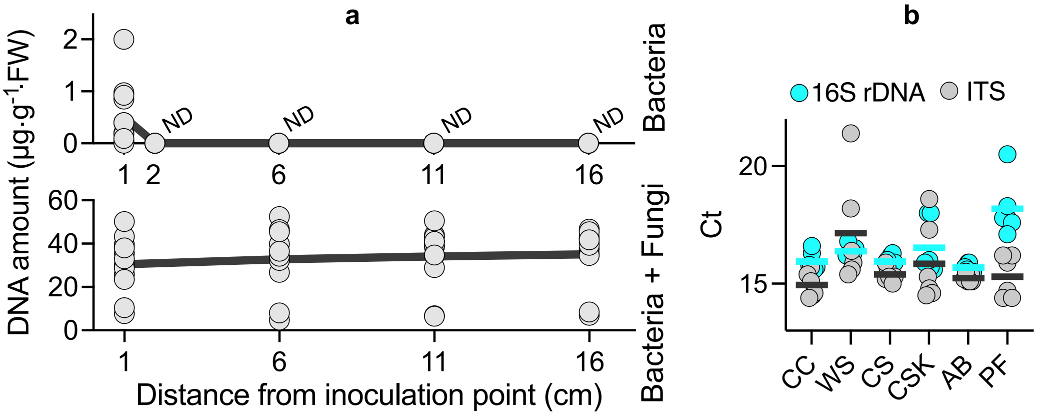

Fig. S7. DNA-related biomass evaluation in the solid-state fermentation of the synthetic microbiota. After 10 days of fermentation, substrates located at 1 cm, 6 cm, 11 cm and 16 cm from the inoculation point were sampled and utilized for DNA extraction to assess microbial biomass (**a**). In the mixed fermentation of bacteria and fungi, fungal and bacterial abundance was evaluated via qPCR quantification of 16S rDNA and ITS (**b**). Lines represent means and ND signifies "not detected".

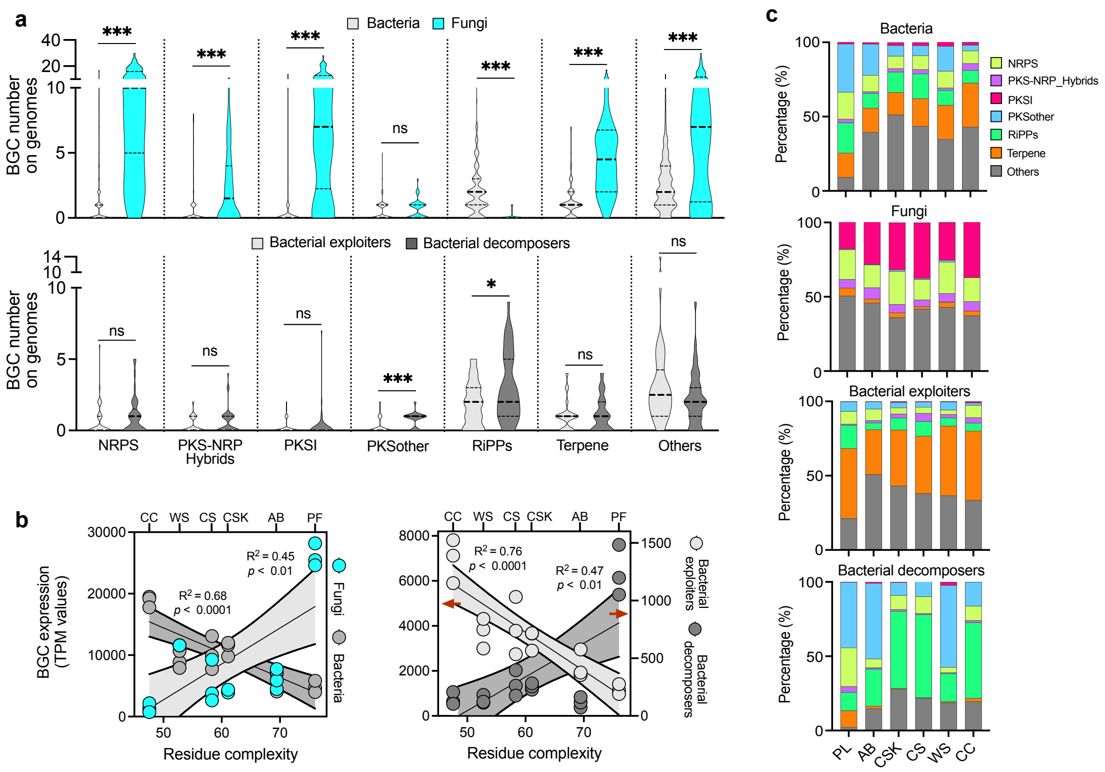

Fig. S8. Analysis of secondary metabolite biosynthetic gene clusters (smBGCs) in fungal and bacterial communities of plant residue decomposition. (**a**) The distribution of smBGC numbers across OTU-matched genomes (Top: 76 fungal genomes and 421 bacterial genomes; Bottom: 43 bacterial exploiter genomes with OTU abundance ≥ 1% and 40 bacterial decomposer genomes with OUT abundance ≤ 0.1‰). (**b**) The relationship between residue complexity and smBGC expression in fungi and bacteria (left), as well as bacterial exploiters and bacterial decomposers (right). (**c**) Proportions of different smBGC categories (NRPS, PKS-NRP Hybrids, PKSI, PKSother, RiPPs, Terpene and Others) expressed within fungi, bacteria, bacterial exploiters and bacterial decomposers across metatranscriptomic samples. The CC, WS, CS, CSK, AB and PF represent each plant residue-incubated metatranscriptomic sample, respectively, with three biological replicates. Asterisks denote statistical significance: *, $p < 0.05$; ***, $p < 0.001$; ns, not significant.

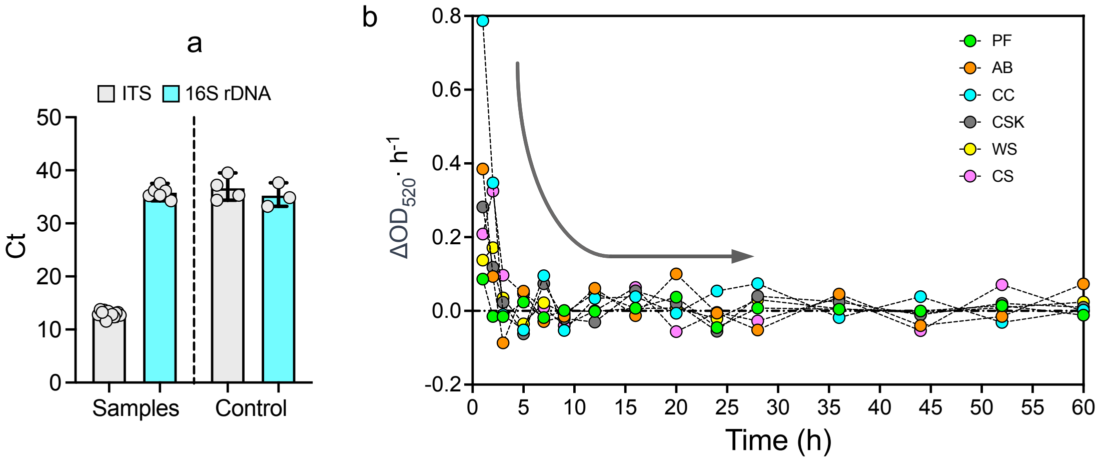

Fig. S9. Bacteria-free confirmation of fungal hyphal tips and hydrolysis analysis. In the mixed fermentation of bacteria and fungi, DNA was extracted from the tips (1-2 cm) of the fungal hyphae in different residue fermentations, then normalized to 5ng·μL^{-1}, and examined using qPCR for bacterial 16S rDNA and fungal ITS, with water as the control (**a**). Detection points without fluorescence curves are not displayed. Equivalent amounts of extracellular enzymes from the fermentation were incubated in liquid hydrolysis experiments at 28 °C for six sterilized plant residues (CC, WS, CS, CSK, AB and PF). Dynamic alterations in reducing sugars release are shown as the incubation time (**b**).
