# Supplementary material for: A novel decomposer-exploiter interaction framework of plant residue microbial decomposition
Source: Genome Biol. 2025 Feb 3;26:20. doi: 10.1186/s13059-025-03486-w (PMC11792400; doi:10.1186/s13059-025-03486-w)
Supplement: Supplementary file 9 — Additional file 9. Review history. [file 13059_2025_3486_MOESM9_ESM.docx]

**1^st^ round**

**Reviewer 1**

Microorganisms are essential for recycling nutrients from complex biomasses such as plant residues on Earth. Fungi and bacteria are capable of decomposing complex substrates, which otherwise cannot be utilized by other organisms, into simpler forms. In nature, plant residues come in diverse complexities, and it is not clear how fungi and bacteria interact and cooperate in the decomposition process and how the microbial community evolves in different plant residues. This work studied the evolution of microbial communities during the decomposition of six plant residues with different hemicellulose, cellulose, and lignin contents. The authors found that plant residue complexity is positively correlated with fungal richness and negatively correlated with bacterial richness. They further analyzed the gene contents of the bacterial and fungal communities using genome analysis and metatranscriptomic sequence analysis. Their results indicate that the fungal communities promote plant residue degradation (decomposers), while the bacterial communities rely on the products released from plant residue decomposition (exploiters). Finally, the authors showed that the presence or absence of fungi can alter the bacterial species enriched in plant residue decomposition, presumably by influencing the carbon acquisition strategy of the bacterial communities.

Understanding the interaction and evolution of microbial communities in plant residue decomposition is of fundamental importance. Although fungi (rather than bacteria) are generally believed to be the main decomposers based on their genome contents (e.g., rich in enzymes for hemicellulose, cellulose, and lignin degradation), this work has provided key experimental evidence to confirm this notion. The experiments were well designed, the results are clearly presented, and the conclusions are justified. Overall, the work addresses an important question and has provided important insights into the interaction and evolution of microbial communities during plant residue decomposition. I think that the work is interesting and novel, and the findings are of general interest to biologists. Therefore, it is suitable for publication in Genome Biology. A few comments to improve the manuscript are listed below.
In the evolution experiment, it is unclear from the statement in lines 93-94, "the same homogenized microbial consortia derived from natural habitats," and the method section what exactly the microbial consortia are. For example, what species are in the consortia before evolution (i.e., at the beginning of the experiment)? If still possible (e.g., the microbial consortia are still available in the lab), this control should be included in the same metatranscriptomic analysis similar to that done at the 14th generation. This will allow a perspective into the microbial community evolution. Alternatively, if information on the microbial composition in the consortia is known from any other means, please provide the information. Otherwise, justification(s) on why the control information is not necessary for the conclusions should be discussed.

Related to this, please describe "the natural habitats" (e.g. temperature range, humidity, plant residue type, etc.) and explain the reason(s) behind choosing these habitats briefly in the results section and in detail in the methods section.

The decomposition experiment was performed at 28˚C. Presumably, the growth rate at this temperature differs among the bacterial and fungal species in the consortia, and so the result may be different in the wild environment where temperature can fluctuate a lot. The authors should discuss the possible (or lack of) effects of temperature on their results and conclusions related to the evolution of microbial communities in plant residue decomposition.

Microorganisms can produce substances (e.g., peptides, secondary metabolites, etc.) for cell-to-cell and species-to-species interaction and to promote or inhibit the growth of other species/organisms. The authors may want to add some discussion on the possible effects of this on the evolution of microbial communities during plant residue decomposition and comment on whether this might have happened in their decomposition experiment. For example, fungi might produce mycotoxins to inhibit grow of certain bacteria that compete for the same nutrients (e.g. decomposer fungi inhibiting decomposer bacteria). If this indeed happened, the observed effect of fungi in changing the decomposer bacteria to exploiter bacteria in the community might not simply be due to carbon availability.

Related to this point. In fact, the authors could use the same metatranscriptomic analysis on secondary metabolite biosynthesis gene clusters to reveal if specific substances are produced by the micro-communities and whether certain metabolites are produced in specific plant residues by examining specific metabolic gene clusters (both known and predicted). Although this analysis is feasible and the results could provide additional insights into the evolution, it may be beyond the scope of this paper. Therefore, this is an optional suggestion.

Line 301: "bacterial strains in-between". Suggest to use another description for this.

Line 389: "This study reveals a groundbreaking interaction between fungi and bacteria in plant residue decomposition." Suggest to replace the word "groundbreaking" in this sentence.

**Reviewer 2**

The manuscript focuses on the analysis of a novel decomposer-exploiter interaction framework emphasizing the specific roles and functions of bacterial and fungal populations.
The applied (meta)genomic and (meta)transcriptomic approaches are fully suitable for this investigation, all important control experiments were executed, the numbers of samples are appropriate to make statistically valid conclusions. All important details are shared, the supprting material is highly useful and explain all aspects.
The study brings sufficient novel information to the research field. The observed interplay between bacteria and fungi in plant residue decomposition is highly interesting. Fungi consistently dominated the decomposer communities, and it was not dependent on plant residue complexity. It is clearly proven, that bacteria mostly utilized fungal degradation products, thus, their basically acted as exploiters. This utilizer-exploiter realtionship is very well described and is supported by statistically relevant data. The figures are highly informative and clear.
The paper will be of interest to microbial ecologists regardless their specific topic.

A few typos were observed, and English can be somewhat improved (there is no major issue with this). So I recommend a final careful reading by a native English speaker.

**Authors’ response**

**Reviewer #1:** Microorganisms are essential for recycling nutrients from complex biomasses such as plant residues on Earth. Fungi and bacteria are capable of decomposing complex substrates, which otherwise cannot be utilized by other organisms, into simpler forms. In nature, plant residues come in diverse complexities, and it is not clear how fungi and bacteria interact and cooperate in the decomposition process and how the microbial community evolves in different plant residues. This work studied the evolution of microbial communities during the decomposition of six plant residues with different hemicellulose, cellulose, and lignin contents. The authors found that plant residue complexity is positively correlated with fungal richness and negatively correlated with bacterial richness. They further analyzed the gene contents of the bacterial and fungal communities using genome analysis and metatranscriptomic sequence analysis. Their results indicate that the fungal communities promote plant residue degradation (decomposers), while the bacterial communities rely on the products released from plant residue decomposition (exploiters). Finally, the authors showed that the presence or absence of fungi can alter the bacterial species enriched in plant residue decomposition, presumably by influencing the carbon acquisition strategy of the bacterial communities.

Understanding the interaction and evolution of microbial communities in plant residue decomposition is of fundamental importance. Although fungi (rather than bacteria) are generally believed to be the main decomposers based on their genome contents (e.g., rich in enzymes for hemicellulose, cellulose, and lignin degradation), this work has provided key experimental evidence to confirm this notion. The experiments were well designed, the results are clearly presented, and the conclusions are justified. Overall, the work addresses an important question and has provided important insights into the interaction and evolution of microbial communities during plant residue decomposition. I think that the work is interesting and novel, and the findings are of general interest to biologists. Therefore, it is suitable for publication in Genome Biology. A few comments to improve the manuscript are listed below.

**Response:** Thank you for your valuable feedback. We have carefully considered your comments and made comprehensive revisions to the manuscript.

In the evolution experiment, it is unclear from the statement in lines 93-94, "the same homogenized microbial consortia derived from natural habitats," and the method section what exactly the microbial consortia are. For example, what species are in the consortia before evolution (i.e., at the beginning of the experiment)? If still possible (e.g., the microbial consortia are still available in the lab), this control should be included in the same metatranscriptomic analysis similar to that done at the 14th generation. This will allow a perspective into the microbial community evolution. Alternatively, if information on the microbial composition in the consortia is known from any other means, please provide the information. Otherwise, justification(s) on why the control information is not necessary for the conclusions should be discussed.

**Response：**Thank you very much for your valuable suggestions. This experiment aimed to develop efficient and stable microbial communities for decomposing plant residues of varying complexity and, on this basis, to explore the microbial interaction mechanisms driving the decomposition under complexity differences. To achieve this, we collected 127 diverse samples, including plant residue compost, significantly decomposed litter from forests or grasslands, and organic matter-rich topsoil, to maximize the potential diversity of plant residue-decomposing microbes. These samples were thoroughly homogenized to serve as the initial inoculum.

Unlike conventional evolution experiments, the primary purpose of the initial inoculum was to provide the broadest possible range of potential microbial resources. However, the initial inoculum itself was an extremely complex, diverse, and unstable artificial microbial community. Introducing such a community into evolutionary analyses could significantly increase complexity and uncertainty. Moreover, plant residue-associated environmental samples are not entirely random microbial communities and exhibit conditioning imprints, making them less suitable as controls for comparative analysis.

Nonetheless, we fully acknowledge the validity of your suggestion. Including information on the initial inoculum could provide additional interesting insights. This was also noted during the analysis stage; however, due to the considerations outlined earlier in the experiment’s planning phase, the mixed initial inoculum was regrettably not preserved. Therefore, this study, under its initial design, focuses on exploring the relationship between plant residue complexity and fungal-bacterial decomposition interactions, based on stable microbial communities evolved from different plant residues. The initial mixed inoculum effectively drove the formation of stable decomposition communities, and while specific data on it is absent, this limitation does not compromise the study’s main conclusions or core findings. Once again, we sincerely appreciate your suggestion and have addressed this point in lines 98-101 and 431-436, providing appropriate explanations.

Related to this, please describe "the natural habitats" (e.g. temperature range, humidity, plant residue type, etc.) and explain the reason(s) behind choosing these habitats briefly in the results section and in detail in the methods section.

**Response：**Thank you very much for your suggestion. We have incorporated detailed explanations into the results and methods sections (lines 98-101 and 457-469).

The decomposition experiment was performed at 28˚C. Presumably, the growth rate at this temperature differs among the bacterial and fungal species in the consortia, and so the result may be different in the wild environment where temperature can fluctuate a lot. The authors should discuss the possible (or lack of) effects of temperature on their results and conclusions related to the evolution of microbial communities in plant residue decomposition.

**Response：**Thank you very much for your valuable suggestion. Temperature significantly influences microbial metabolism and growth rates and plays a crucial role in determining plant residue turnover rates and soil organic matter accumulation efficiency within global ecological systems. Therefore, it is indeed essential to consider the impact of temperature on plant residue decomposition communities. Additionally, other environmental factors, such as pH, salinity and moisture content, may also affect these communities. We have included additional discussions on this topic in lines 431 to 445.

Microorganisms can produce substances (e.g., peptides, secondary metabolites, etc.) for cell-to-cell and species-to-species interaction and to promote or inhibit the growth of other species/organisms. The authors may want to add some discussion on the possible effects of this on the evolution of microbial communities during plant residue decomposition and comment on whether this might have happened in their decomposition experiment. For example, fungi might produce mycotoxins to inhibit grow of certain bacteria that compete for the same nutrients (e.g. decomposer fungi inhibiting decomposer bacteria). If this indeed happened, the observed effect of fungi in changing the decomposer bacteria to exploiter bacteria in the community might not simply be due to carbon availability.

Related to this point. In fact, the authors could use the same metatranscriptomic analysis on secondary metabolite biosynthesis gene clusters to reveal if specific substances are produced by the micro-communities and whether certain metabolites are produced in specific plant residues by examining specific metabolic gene clusters (both known and predicted). Although this analysis is feasible and the results could provide additional insights into the evolution, it may be beyond the scope of this paper. Therefore, this is an optional suggestion.

**Response：**Thank you very much for your suggestion. Antagonistic competition is an inherent feature of microbial communities. The distinct functional roles observed in plant residue decomposition—characterized by “fungal decomposers-bacterial exploiters”—highlight the significant differentiation between fungal decomposers, bacterial exploiters and bacterial decomposers, suggesting the presence of intense competitive interactions. Follow your suggestion, we conducted a comprehensive analysis of the distribution and expression patterns of fungal and bacterial secondary metabolite biosynthetic gene clusters (smBGCs), uncovering additional key evidence supporting the dynamic interplay between fungi and bacteria.

Our results showed that bacterial smBGC expression was significantly negatively correlated with plant residue complexity (R^2^ = 0.68，*p* < 0.0001), consistent with the ecological understanding that competition is stronger under nutrient-rich conditions and weaker under nutrient-poor conditions. In contrast, fungal smBGC expression exhibited a significant positive correlation with residue complexity (R^2^ = 0.45，*p* < 0.01), suggesting that competition among fungi intensifies as nutrient availability declines. This new insight explains the observed increase in fungal richness and the corresponding sharp decline in bacterial richness with increasing residue complexity. The sharp decline in bacterial exploiter smBGC expression reflects a strategic compromise in competition, as exploiters rely on fungal decomposers for survival under such conditions.

Further analysis revealed a strong negative correlation between bacterial exploiter smBGC expression and residue complexity (R^2^ = 0.76，*p* < 0.0001), consistent with the dominance of exploiters within bacterial communities. Interestingly, bacterial decomposers displayed a markedly different pattern, with their smBGC expression significantly positively correlated with residue complexity (R^2^ = 0.47, *p* < 0.01), mirroring the trend observed for fungal decomposers. Bacterial decomposers share an ecological niche with fungal decomposers, which determines their similar external competitive strategies while also implying intense competition between the two groups. In addition, we performed comparative analyses of smBGC classifications and potential products, revealing distinct expression patterns among fungal decomposers, bacterial decomposers and bacterial exploiters. These findings are discussed in detail in the Discussion sections of the manuscript (lines 352-401). Thank you for this valuable suggestion, which has greatly enriched our study.

Line 301: "bacterial strains in-between". Suggest to use another description for this.

**Response：**Thanks, it has been changed to “intermediate bacterial strains” throughout the manuscript.

Line 389: "This study reveals a groundbreaking interaction between fungi and bacteria in plant residue decomposition." Suggest to replace the word "groundbreaking" in this sentence.

**Response：**Thank you for pointing this out. The overly descriptive term has been replaced with “remarkable” in the manuscript, as suggested.

**Reviewer #2:** The manuscript focuses on the analysis of a novel decomposer-exploiter interaction framework emphasizing the specific roles and functions of bacterial and fungal populations.

The applied (meta)genomic and (meta)transcriptomic approaches are fully suitable for this investigation, all important control experiments were executed, the numbers of samples are appropriate to make statistically valid conclusions. All important details are shared, the supporting material is highly useful and explain all aspects.

The study brings sufficient novel information to the research field. The observed interplay between bacteria and fungi in plant residue decomposition is highly interesting. Fungi consistently dominated the decomposer communities, and it was not dependent on plant residue complexity. It is clearly proven, that bacteria mostly utilized fungal degradation products, thus, their basically acted as exploiters. This utilizer-exploiter relationship is very well described and is supported by statistically relevant data. The figures are highly informative and clear.

The paper will be of interest to microbial ecologists regardless their specific topic.

**Response：**We sincerely thank you for your review and recognition of our manuscript. Your positive evaluation is highly encouraging.

A few typos were observed, and English can be somewhat improved (there is no major issue with this). So I recommend a final careful reading by a native English speaker.

**Response：**Thank you very much. We have carefully checked the whole manuscript and made corrections to any potential misunderstandings or typos.

**2^nd^ round**

**Reviewer 1**

The authors have addressed my comments. Congratulations.
